# Development and validation of early-stage and progression prediction models for chronic kidney disease: a retrospective study

**DOI:** 10.7717/peerj.20931

**Published:** 2026-03-16

**Authors:** Tongyuan Wan, Qi Chen, Yiming Gao, Renli Luo, Nan Li, Yonghui Feng

**Affiliations:** 1 National Clinical Research Center for Laboratory Medicine, Department of Laboratory Medicine, The First Hospital of China Medical University, Shenyang, China; 2 State Key Laboratory for Diagnosis and Treatment of Infectious Diseases, NHC Key Laboratory of AIDS Prevention and Treatment, National Clinical Research Center for Laboratory Medicine, The First Hospital of China Medical University, China Medical University, Shenyang, China

**Keywords:** Chronic kidney disease, Nomogram, Predictive model, Risk factors, Prognosis, Early-stage prediction

## Abstract

**Objectives:**

Chronic kidney disease (CKD) poses a significant public health burden. This study aimed to evaluate the associations between clinical laboratory indices and CKD and to develop prediction and prognostic models for CKD risk assessment and disease progression.

**Design & Methods:**

Between January 2008 and June 2018, we enrolled 500 healthy controls, 445 patients with early-stage CKD (G1–G2), and 527 patients with CKD G5 at the First Hospital of China Medical University. Logistic regression analyses were performed to identify independent predictors for the presence of CKD and progression to advanced disease, which were subsequently incorporated into visual nomograms. Model performance was evaluated using area under the receiver operating characteristic curves (AUC) and calibration plots. Clinical utility was assessed using decision curve analysis (DCA) and clinical impact curves (CIC).

**Results:**

The early-stage CKD prediction nomogram achieved an AUC of 0.981 in the training set and 0.969 in the validation set. The progression nomogram demonstrated AUC values of 0.984 and 0.972 in the training and validation sets, respectively. DCA and CIC analyses further confirmed the clinical relevance and potential applicability of both models.

**Conclusions:**

We developed and validated early-stage prediction and progression assessment for CKD, demonstrating high discriminative ability, good calibration, and significant clinical utility. These models may facilitate early detection and dynamic risk assessment in CKD management.

## Introduction

Chronic kidney disease (CKD) is a common and progressive disorder characterized by persistent abnormalities in renal structure and/or function arising from multiple pathological pathways (Webster et al., 2017; GBD Chronic Kidney Disease Collaboration, 2020). CKD is associated with a wide range of adverse outcomes, such as kidney failure, cardiovascular disorders, and increased mortality. Consequently, it represents a major public health challenge (GBD 2017 Causes of Death Collaborators, 2018). According to the Kidney Disease Improving Global Outcomes (KDIGO) guidelines, CKD is classified into five stages (G1–G5) based on glomerular filtration rate (GFR) (GBD 2019 Risk Factors Collaborators, 2020). However, due to the absence of specific clinical manifestations, the detection rate of early-stage CKD remains low, reported to be less than 5% (Chen, Knicely & Grams, 2019). As the disease progresses, many patients require renal replacement therapy, leading to a deterioration in quality of life. Therefore, early identification of individuals at risk for CKD and early-stage prediction for disease progression are critical for preventing adverse clinical outcomes.

Clinical laboratory indicators are essential for monitoring the disease progression and provide information regarding renal function. Serum creatinine (Cr), a metabolite of muscle turnover, is a widely used marker of kidney function and forms the basis of estimated GFR calculation in the CKD-EPI equation when combined with age and sex (Yang et al., 2020). Urea and urinary albumin concentration are related to urine osmotic pressure or maximal urine concentration, which directly reflect kidney function and assist clinical risk stratification (Weaver, 2019). Given that CKD can cause systemic multisystem involvement (Romagnani et al., 2017), parameters beyond traditional renal indices are also worthy of attention. For example, CKD has been consistently associated with abnormal hemoglobin (Hb) (Guedes et al., 2020), resulting from decreased erythropoietin or reduced iron absorption (Atkinson & Warady, 2018; Nakanishi, Kimura & Kuragano, 2019). Moreover, abnormal glucose metabolism, lipid metabolism and coagulation function have been reported in CKD, manifesting as hyper-coagulation, bleeding tendency, or metabolic dysregulation (Dincer et al., 2019). Therefore, metabolic and coagulation indicators may contribute to risk assessment and disease treatment.

Although individual clinical indicators provide important clinical insights, reliance on a single parameter has limited predictive value and clinical applicability. In contrast, multivariable prediction models are suitable for risk stratification and clinical decision support. Currently, some developed predictive models are used to predict the progression of CKD. A predictive model for the dynamic monitoring of CKD changes was established by Tangri et al. (2017) through the utilization of conventional clinical testing indicators. In addition, a prediction model for CKD at an advanced stage after acute kidney injury was devised by James et al. (2017). Although several CKD models already exist, no model can simultaneously predict disease risk and prognosis.

In the present study, we screened commonly available laboratory indicators and developed two complementary prediction models. A diagnostic model incorporating renal function indices, coagulation indices, and metabolic indices was constructed to estimate the risk of early-stage CKD. Subsequently, a prognostic model was established to predict progression from G1–G2 to G5. We developed a prognostic nomogram based on five indicators to predict the risk of progression from early-stage CKD to G5. Nomograms were employed to visualize both models, facilitating clinical implementation. The models demonstrated satisfactory discrimination, calibration, and clinical utility, supporting their potential value in early detection and individualized risk assessment of CKD.

## Materials and Methods

### Study population

We conducted a retrospective collection of all clinical data from patients who either made their first visit or underwent a physical examination at the First Hospital of China Medical University between January 2008 and June 2018. CKD was defined in accordance with the Kidney Disease: Improving Global Outcomes (KDIGO) 2023/2024 guidelines as the presence of abnormalities in kidney structure or function persisting for at least 3 months.

Evidence of kidney damage included one or more of the following criteria: histological abnormalities identified on renal pathology; persistent abnormalities in laboratory parameters, such as increased urine albumin-to-creatinine ratio (≥30 mg/g), elevated 24-h urinary albumin excretion (≥30 mg), increased total urinary protein, or presence of nephrolithiasis, or cystic changes. A sustained reduction in estimated glomerular filtration rate (eGFR) to <60 mL/min/1.73 m^2^ for at least 3 months was indicative of glomerular dysfunction; and structural abnormalities detected by imaging, including renal atrophy, altered renal morphology, are considered sufficient for CKD classification irrespective of other findings. In participants with an eGFR of 60–89 mL/min/1.73 m^2^, CKD was diagnosed only when accompanied by additional evidence of kidney damage as defined above (Kelly et al., 2021; Al Khalaf et al., 2022). Individuals with isolated eGFR values in this range without corroborating markers of kidney damage were not classified as having CKD.

Exclusion criteria were as follows: (1) initiation of dialysis at the first visit; (2) history of kidney transplantation prior to enrollment; and (3) missing clinical data exceeding 50%. Based on these criteria, 445 patients with early-stage CKD (G1–G2) and 500 age- and sex-matched healthy controls were included to develop an early-stage CKD prediction nomogram. To establish the progression model, we further analyzed data from 527 patients with CKD G5 in conjunction with the early-stage CKD cohort.

Given the retrospective design, anonymization of patient data, and previously collected clinical data, the requirement for written informed consent was waived. This study was conducted in accordance with the Declaration of Helsinki and was approved by the Ethics Committee of the First Hospital of China Medical University (approval number: 2020-323).

### Data collection

Demographic information, including age and sex, together with laboratory measurements from blood and urine samples, were retrieved from the institutional database. Serum levels of creatinine (Cr), cystatin C (Cys), urea, C-reactive protein (CRP), uric acid (UA), phosphorus (P), calcium (Ca), total cholesterol (TC), triglycerides (TG), low-density lipoprotein cholesterol (LDL-C), and high-density lipoprotein cholesterol (HDL-C) were measured using an automated biochemical analyzer (Hitachi 7600, Hitachi, Tokyo, Japan). Plasma fibrinogen (FIB) was assessed with the STA-R Evolution system (Diagnostica Stago, Asnières-sur-Seine, France). Hemoglobin (Hb) concentrations were determined using the Sysmex XN-20 hematology analyzer (Sysmex, Kobe, Japan), and urinary microalbumin (mALB) levels were quantified with the Siemens BN II nephelometer (Siemens, Munich, Germany).

### Statistical analysis

#### Prediction model development

Based on the normality assessment with the Kolmogorov-Smirnov test, we presented continuous variables as either means ± standard deviation or medians with interquartile ranges. We compared variables between groups using either Student’s independent t-test or the Mann-Whitney U test. Categorical variables were presented as absolute counts along with their relative frequencies and were analyzed using the chi-square test. Continuous predictors were standardized by subtracting the mean and dividing by the standard deviation, or scaled using clinically meaningful units, to ensure odds ratios are interpretable.

Due to the presence of missing data for some parameters, multiple imputation was performed using the chained equations (MICE) method, assuming missing at random. Ten imputed datasets were generated, and analyses were conducted by pooling results across the imputed datasets. The complete datasets were randomly divided into a training set and a validation set at a 7:3 ratio. To ensure robustness, bootstrap resampling was applied, allowing each observation an equal probability of inclusion into the training or validation set. Sixteen candidate variables were initially considered based on clinical relevance and published literature. Univariate logistic regression identified potential predictors of CKD incidence or progression. Multicollinearity was evaluated using the variance inflation factor (VIF), with VIF ≤4 considered acceptable. Variables were further refined using backward stepwise multivariate logistic regression. Variables such as cystatin C and urinary albumin concentration were excluded due to poor model fit or nonsignificant associations. Importantly, selection was guided by both statistical criteria and clinical applicability, prioritizing variables that are routinely measured and interpretable in clinical practice. Independent predictors identified from the training set were incorporated into visual nomograms for early-stage CKD diagnosis and progression prediction to advanced CKD. Model performance was assessed using receiver operating characteristic (ROC) curves, area under the ROC curve (AUC), and calibration plots, supplemented with the Hosmer-Lemeshow test. Clinical utility was evaluated using decision curve analysis (DCA) and clinical impact curves (CIC) for a hypothetical population of 1,000 patients. All statistical analyses were conducted using R software (version 4.1.1), with a two-sided *p*-value < 0.05 considered statistically significant.

#### Evaluation of model performance

To evaluate the value of the models, we conducted a series of performance validations and assessments on the established nomograms in both the training and validation sets. We assessed the discriminatory power of the nomograms using the receiver operating characteristic (ROC) curve and quantified it with the AUC. Calibration curves were utilized to determine the consistency of the nomogram, and the Hosmer-Lemeshow (H-L) test was performed. Specifically, the decision curve analysis (DCA), which charts the net benefit (NB) across a range of risk thresholds aligned with clinical practice, was employed to evaluate the clinical utility of the nomogram for a population of 1,000 individuals. Furthermore, clinical impact curves (CIC) were devised on the basis of the decision curve analysis (DCA). These curves aimed to vividly display the approximated number of high-risk patients at every risk threshold.

## Results

### Characteristics of the early-stage CKD prediction model

**Figure 1:**
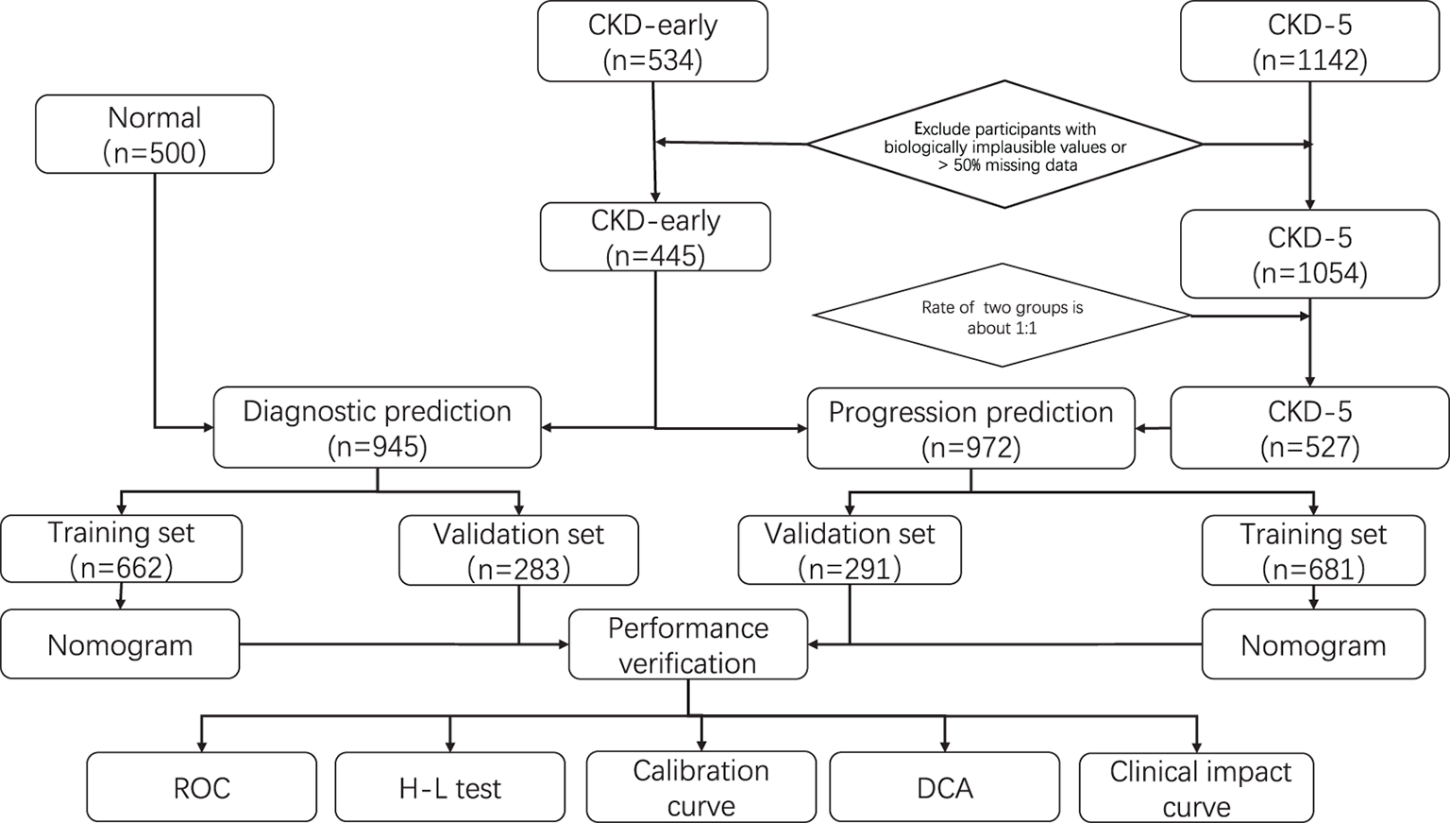
Flowchart of the procedure. Data from 534 patients with early CKD, 1,142 patients with stage 5 CKD, and 500 sex-and-age-matched healthy controls were included in our study. After screening and matching, the 445 early CKD data were used to establish the early-stage prediction nomogram with 500 healthy controls and progression nomogram with 527 advanced CKD patients respectively.

Figure 1 illustrates a flowchart that elucidated the entire process of the study. The comparison of clinical characteristics between early-stage CKD patients and healthy controls was shown in Table 1. The early CKD patients had higher Cr, FIB, Cys, UA, Urea, P, TC, TG, LDL, and mALB (all *p*-values < 0.001) and lower Ca, HDL, and Hb (all *p*-values* *< 0.001) compared with healthy controls. Moreover, no statistically significant differences in gender, age, and CRP were detected between the two groups (*p*-value *>* 0.05). The same results were also noted in the training set (*n* = 662) and validation set (*n* = 283), which are presented in Table 2.

**Table 1:** Basic characteristics of laboratory indicators for patients in CKD stage 1 to 2 and normal population.

Indicators	Early	Normal	*p* value
	(*n* = 445)	(*n* = 500)	
Gender Male, *n* (%)	290 (65%)	325 (65%)	1
Female, *n* (%)	155 (35%)	175 (35%)	
Age (years)	51 (38, 62)	51 (38, 62)	0.73
Cr (μmol/L)	105 (85, 121)	61 (53, 71)	<2.2e−16
FIB (g/L)	3.74 (3.21, 4.37)	3.17 (2.82, 3.5025)	<2.2e−16
CRP (mg/L)	2.71 (1.82, 4.58)	2.7 (2.1, 3.5)	0.2
Cys (mg/L)	1.48 (0.45)	0.8 (0.09)	<2.2e−16
UA (μmol/L)	401 (104.7)	299 (62)	<2.2e−16
Urea (mmol/L)	6.94 (5.54, 8.56)	4.945 (4.08, 5.8125)	<2.2e−16
P (mmol/L)	1.17 (0.19)	1.09 (0.15)	7.6e−13
Ca (mmol/L)	2.20 (2.09, 2.28)	2.30 (2.23, 2.35)	<2.2e−16
TC (mmol/L)	4.99 (4.22, 5.99)	4.415 (4.0075, 4.8525)	<2.2e−16
TG (mmol/L)	1.82 (1.26, 2.87)	0.92 (0.69, 1.27)	<2.2e−16
LDL (mmol/L)	3.20 (2.53, 4.10)	2.64 (2.2675, 3.00)	<2.2e−16
HDL (mmol/L)	1.07 (0.90, 1.34)	1.39 (1.18, 1.6625)	<2.2e−16
Hb (g/L)	133 (118, 146)	140 (132, 150)	<2.2e−16
mALB (mg/L)	980 (309, 2,070)	10.60 (10.60, 11.85)	<2.2e−16

**Note:**

Normal-distribution measurement data were expressed as mean ± standard deviation, non-normal distributions are represented by median (P25%, P75%), and counting data were expressed as frequency and percentage, *p* < 0.05 was considered statistically significant. Cr, Creatinine; FIB, fibrinogen; CRP, C-reactive protein; Cys, Cystatin C; UA, serum uric acid; Urea, urea nitrogen; P, serum phosphorus; Ca, serum calcium; TC, total cholesterol; TG, triglyceride; LDL, low density lipoprotein; HDL, high-density lipoprotein; Hb, hemoglobin; mALB, microalbuminuria.

**Table 2:** Data comparison in the training and validation group of the diagnostic prediction model.

Indicators		Train			Validation	
		(*n* = 662)			(*n* = 283)	
	Early	Normal	*p* value	Early	Normal	*p* value
	(*n* = 305)	(*n* = 357)		(*n* = 140)	(*n* = 143)	
Gender Male, *n* (%)	199 (65%)	234 (66%)	1	91 (65%)	91 (64%)	0.91
Female, *n* (%)	106 (35%)	123 (34%)		49 (35%)	52 (36%)	
Age (years)	50 (38, 62)	51 (39, 62)	0.63	53.5 (39, 63)	49 (38, 61)	0.19
Cr (μmol/L)	106 (88, 121)	61 (54, 71)	<2.2e−16	105 (77.75, 121)	60 (52, 70)	<2.2e−16
FIB (g/L)	3.68 (3.26, 4.33)	3.16 (2.82, 3.51)	<2.2e−16	3.805 (3.1175, 4.6125)	3.21 (2.77, 3.48)	2.1e−10
CRP (mg/L)	2.70 (0.90, 4.36)	2.70 (2.2, 3.55)	0.76	2.755 (1.8075, 4.8225)	2.6 (2.0, 3.3)	0.095
Cys (mg/L)	1.47 (1.18, 1.74)	0.8 (0.73, 0.87)	<2.2e−16	1.42 (1.08, 1.73)	0.8 (0.74, 0.87)	<2.2e−16
UA (μmol/L)	397 (334, 477)	299 (257, 348)	<2.2e−16	379 (320.25, 465.75)	285 (246.5, 345.5)	3.7e−16
Urea (mmol/L)	7.11 (5.68, 8.65)	4.96 (4.19, 5.84)	<2.2e−16	6.72 (5.2375, 8.225)	4.9 (3.89, 5.72)	3.5e−16
P (mmol/L)	1.16 (1.06, 1.28)	1.09 (0.99, 1.18)	8.9e−10	1.16 (1.0575, 1.3025)	1.08 (0.955, 1.215)	0.0003
Ca (mmol/L)	2.21 (2.09, 2.29)	2.3 (2.23, 2.36)	<2.2e−16	2.175 (2.06, 2.26)	2.29 (2.23, 2.34)	<2.2e−16
TC (mmol/L)	5.08 (4.22, 5.99)	4.43 (4.02, 4.87)	7.3e−14	4.88 (4.2175, 5.9125)	4.33 (3.98, 4.78)	6.9e−7
TG (mmol/L)	1.89 (1.29, 2.94)	0.91 (0.69, 1.27)	<2.2e−16	1.69 (1.165, 2.615)	0.97 (0.73, 1.265)	<2.2e−16
LDL (mmol/L)	3.20 (2.52, 4.15)	2.66 (2.29, 3.01)	<2.2e−16	3.2 (2.6375, 3.9075)	2.58 (2.175, 2.965)	2.3e−11
HDL (mmol/L)	1.06 (0.89, 1.34)	1.39 (1.18, 1.66)	<2.2e−16	1.105 (0.94, 1.33)	1.41 (1.18, 1.69)	9.5e−13
Hb (g/L)	132 (118, 147)	140 (132, 149)	6.1e−10	134 (118, 143.25)	140 (131, 150)	0.0001
mALB (mg/L)	901 (296, 2,060)	10.6 (10.6, 12.1)	<2.2e−16	1045 (338, 2,102.5)	10.6 (10.5, 11.5)	<2.2e−16

**Note:**

Normal-distribution measurement data were expressed as mean ± standard deviation, non-normal distributions are represented by median (P25%, P75%), and counting data were expressed as frequency and percentage, *p* < 0.05 was considered statistically significant. Cr, Creatinine; FIB, fibrinogen; CRP, C-reactive protein; Cys, Cystatin C; UA, serum uric acid; Urea, urea nitrogen; P, serum phosphorus; Ca, serum calcium; TC, total cholesterol; TG, triglyceride; LDL, low density lipoprotein; HDL, high-density lipoprotein; Hb, hemoglobin; mALB, microalbuminuria.

### Establishment of early-stage CKD prediction model

Cr, FIB, Cys, UA, Urea, P, TC, TG, LDL, mALB, Ca, HDL, and Hb were potential predictive factors for early CKD and were included in univariate logistic regression analysis. The results showed that Cr, FIB, UA, Urea, P, TC, TG, LDL, Ca, HDL, and Hb obtained from the training set were associated with CKD incidence, while the Cys and mALB indicators were excluded due to poor fit in Table 3. It is worth mentioning that four indicators of blood fats (TC, TG, LDL, HDL) were found to exhibit multicollinearity by comparing the VIF values. To obtain the prediction model with the best early-stage prediction accuracy, we evaluated five models with all four indicators excluded and retained, respectively. The model retaining TG exhibited the highest AUC value in both the training set (AUC, 0.981, 95% CI [0.972–0.991]) and the validation set (AUC, 0.969, 95% CI [0.951–0.987]), leading to its final selection. Multivariate analysis revealed that Cr (OR, 1.10, 95% CI [1.08–1.13]), FIB (OR, 2.02, 95% CI [1.27–3.42]), UA (OR, 1.01, 95% CI [1.00–1.01]), Ca (OR, 1.12, 95% CI [1.05–1.20]), P (OR, 19.20, 95% CI [2.09–188.80]), TG (OR, 9.50, 95% CI [5.40–18.20]), and Hb (OR, 0.94, 95% CI [0.91–0.96]) were independent predictive factors for early CKD in Table 3.

**Table 3:** Data analysis results of CKD diagnostic prediction model.

Variable	Scaling/Unit	OR (95% CI)	*p* value	VIF
Creatinine (Cr)	per 10 μmol/L increase	1.10 [1.08–1.13]	<0.001	2.30
Fibrinogen (FIB)	per SD increase	2.02 [1.27–3.42]	0.005	1.46
Uric acid (UA)	per 50 μmol/L increase	1.01 [1.00–1.01]	0.008	1.87
Phosphorus (P)	per 0.1 mmol/L increase	1.92 [1.21–3.05]	0.010	1.12
Calcium (Ca)	per 0.1 mmol/L increase	1.12 [1.05–1.20]	<0.001	1.50
Triglycerides (TG)	per 1 mmol/L increase	1.95 [1.54–2.32]	<0.001	5.47
Hemoglobin (Hb)	per 10 g/L increase	0.94 [0.91–0.96]	<0.001	1.43

**Note:**

Continuous variables were rescaled using clinically meaningful units or standardized (per SD) to improve interpretability of odds ratios. Variables with poor model fit or significant multicollinearity (*e.g*., CRP, cystatin C, microalbuminuria concentration, TC, LDL, HDL) were excluded during stepwise selection based on both statistical performance and clinical considerations. ORs represent the relative change in odds per specified unit increase. OR, Odds ratio; CI, confidence interval; Cr, creatinine; FIB, fibrinogen; CRP, C-reactive protein; Cys, Cystatin C; UA, serum uric acid; Urea, urea nitrogen; P, serum phosphorus; Ca, serum calcium; TC, total cholesterol; TG, triglyceride; LDL, low density lipoprotein; HDL, high-density lipoprotein; Hb, hemoglobin; mALB, microalbuminuria.

**Figure 2:**
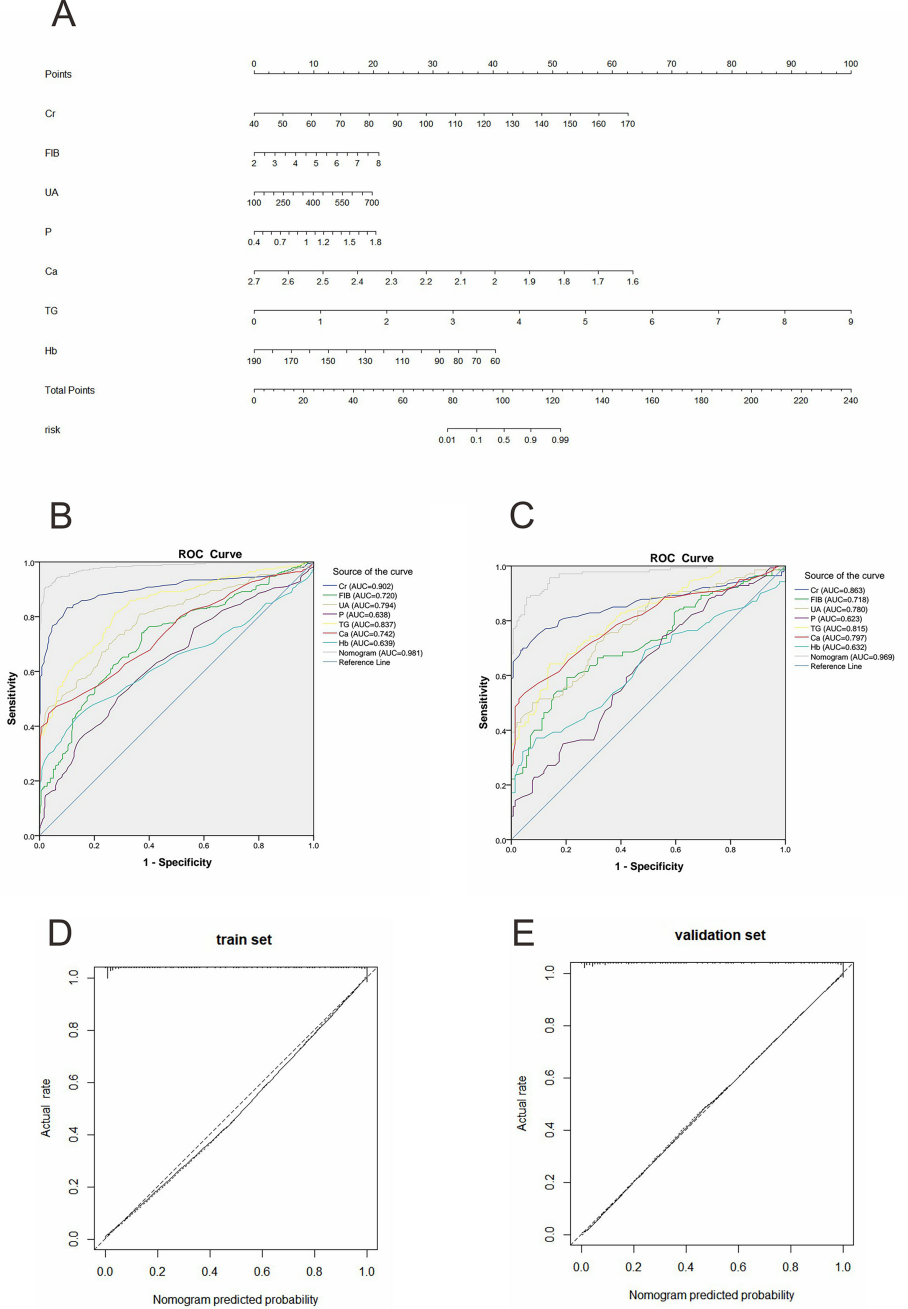
Early-stage CKD prediction model and performance verification. (A) Nomogram of early-stage CKD prediction. Each predictor is assigned a score on the “Points” scale. The total points sum corresponds to the predicted risk of CKD or CKD progression on the “Risk” scale. (B, C) ROC curves of the factors and the nomogram in the training and validation sets, respectively. ROC curves illustrate the discriminative ability of the nomogram; calibration curves show agreement between predicted and observed risks. (D, E) The calibration curves and the reference line of the early-stage CKD prediction model in the training and validation sets, respectively. Cr, creatinine; FIB, fibrinogen; UA, serum uric acid; P, serum phosphorus; Ca, serum calcium; TG, triglycerides; Hb, hemoglobin.

From the outcomes of logistic regression analyses, we utilized Cr, FIB, UA, Ca, P, TG, and Hb to formulate the early CKD nomogram depicted in Fig. 2A. For the application of this nomogram, one should draw a vertical line up to the uppermost point row to ascertain the value of each factor for the specific patient. Then, we calculate the total score by aggregating all individual scores. Finally, draw another vertical line downwards to the bottom row to yield the prediction of the early-stage CKD risk.

### Performance and clinical utility of early-stage CKD prediction model

We evaluated the performance of the nomogram using the AUC, calibration plots, and H-L test in both the training and validation sets. We then constructed the ROC curves for the factors, and the nomogram, with the AUC values indicated (Figs. 2B and 2C). In the training dataset, the AUC of the nomogram was 0.981 (95% CI [0.972–0.991]). In the validation dataset, the AUC was 0.969 (95% CI [0.951–0.987]). These values demonstrated the model’s high discriminatory power. Calibration graphs confirmed an excellent concordance between the predicted risk and the actual observations in both the training and validation datasets, thus revealing good model consistency (Figs. 2D and 2E). Furthermore, the H-L test evaluated the goodness-of-fit of the nomogram. The H-L test chi-square values were 8.152 (*p* = 0.418) and 3.141 (*p* = 0.925) for the training and validation sets, indicating a satisfactory fit between the predicted risk and the actual outcome.

**Figure 3:**
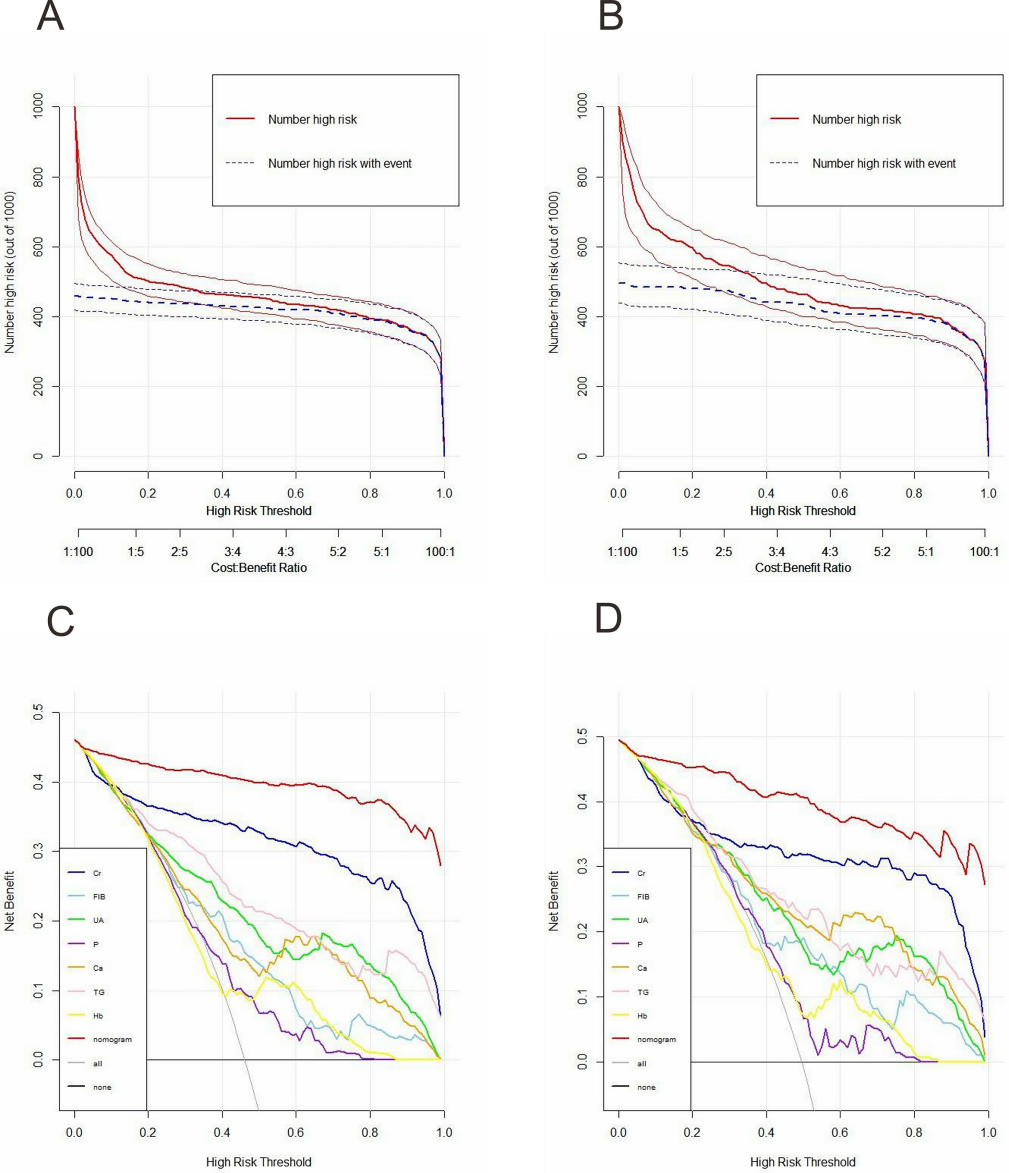
Clinical impact curves (CIC) and decision curve analysis (DCA) for the early-stage prediction model in the training set and validation set. (A) CIC of the early-stage prediction model in the training set. X-axis: predicted risk threshold for CKD; Y-axis: number of patients identified as high-risk per 1,000; The shaded area represents the number of true positives at each threshold. (B) CIC of the early-stage prediction model in the validation set. (C) DCA of the early-stage prediction model in the training set. X-axis: threshold probability for intervention; Y-axis: net benefit per 1,000 patients; the curves compare the net benefit of using the nomogram to strategies of treating all or no patients. (D) DCA of the early-stage prediction model in the validation set.

To explore the clinical applicability of the model’s performance in greater detail, DCA was utilized. The DCA results showed that, within the training set, the net benefit of the nomogram was greater than that of any individual factor (Fig. 3C). Additionally, an analogous outcome was observed in the validation set (Fig. 3D). Additionally, we developed the CIC to evaluate clinical utility. This development relied on the results of the DCA. The CIC indicated that the nomogram achieved acceptable cost-benefit ratios in both the training and validation sets (Figs. 3A and 3B).

### Establishment of progression prediction model

**Figure 4:**
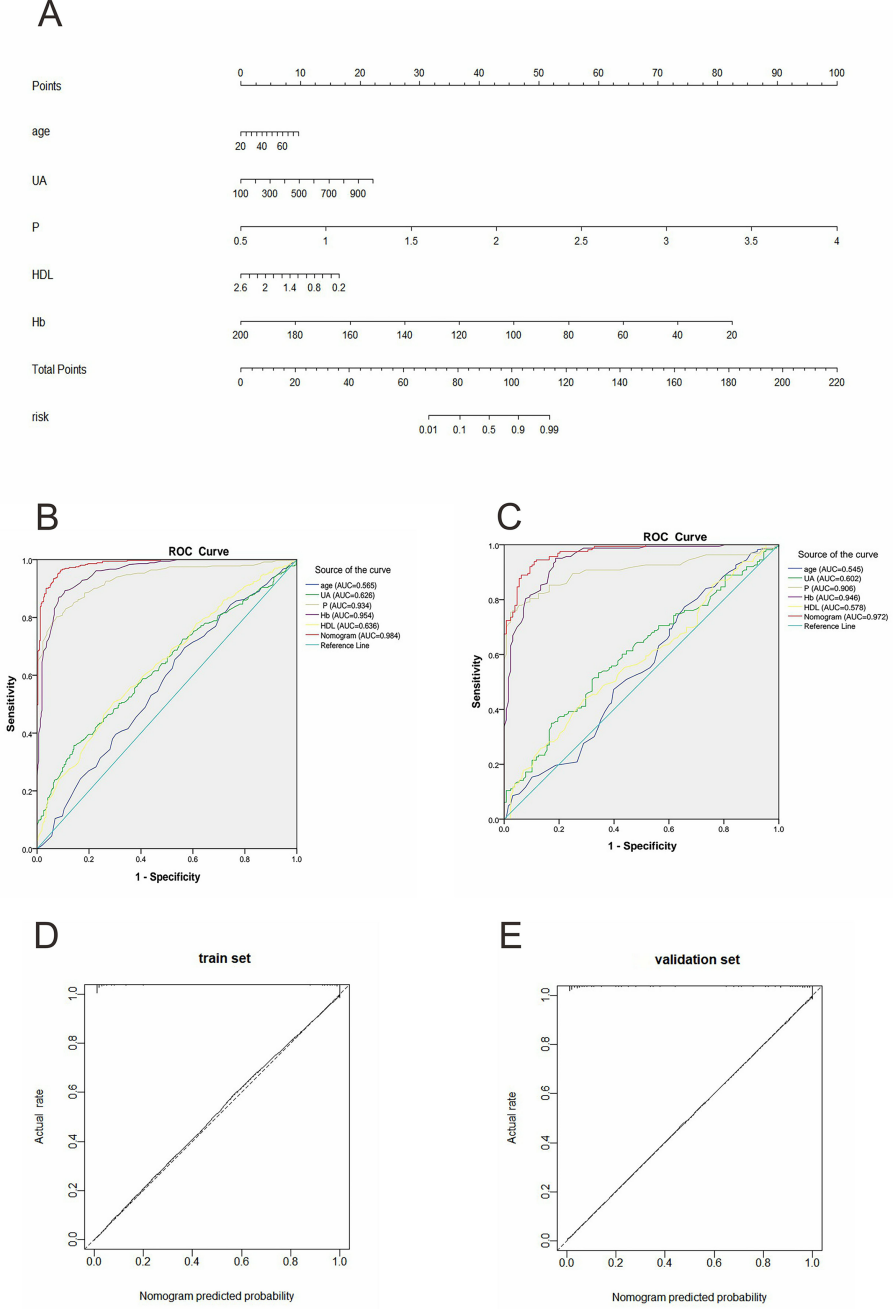
CKD progression prediction model and performance verification. (A) Nomogram of CKD progression prediction model. Each predictor is assigned a score on the “Points” scale. The total points sum corresponds to the predicted risk of CKD or CKD progression on the “Risk” scale. (B) ROC curves of the factors and the nomogram in the training set. ROC curves illustrate the discriminative ability of the nomogram; calibration curves show agreement between predicted and observed risks. (C) ROC curves of the factors and the nomogram in the validation set. (D) The calibration curves and the virtual curve of the progression prediction model in the training set. (E) The calibration curves and the virtual curve of the progression prediction model in the validation set. UA, serum uric acid; P, serum phosphorus; Hb, hemoglobin; HDL, high-density lipoprotein.

Table 4 displays the characteristics of early CKD and CKD G5 patients, while Table 5 presents a comparison of data between the training and validation sets. The data showed that all the included indicators except mALB were statistically different between the two groups, and CKD G5 patients had higher Cr, FIB, CRP, Cys, UA, Urea, and P, and lower Ca, TC, TG, LDL, HDL and Hb (all *p*-values < 0.05) compared with the early-stage CKD. The five indicators, age, UA, P, HDL, and Hb, were proven to be predictors from early-stage CKD to G5 by Univariate, Multicollinearity, and Multivariate analysis (Table 6). The nomogram of the progression prediction model based on these five factors was developed in Fig. 4A.

**Table 4:** Basic characteristics of laboratory indicators for patients in CKD early and CKD-5.

Indicators	CKD-5	Early	*p* value
	(*n* = 527)	(*n* = 445)	
Gender Male, *n* (%)	300 (57%)	290 (65%)	0.009
Female, *n* (%)	227 (43%)	155 (35%)	
Age (years)	54 (42, 63.5)	51 (38, 62)	0.001
Cr (μmol/L)	595 (455, 829.5)	105 (85, 121)	<2.2e−16
FIB (g/L)	4.52 (3.77, 5.38)	3.74 (3.21, 4.37)	<2.2e−16
CRP (mg/L)	4.15 (2.21, 9.10)	2.71 (1.82, 4.58)	6.3e−10
Cys (mg/L)	4.8 (1.1)	1.48 (0.45)	<2.2e−16
UA (μmol/L)	453.9 (139)	401 (104.7)	3e−10
Urea (mmol/L)	26.28 (19.76, 33.48)	6.94 (5.54, 8.56)	<2.2e−16
P (mmol/L)	1.82 (1.54, 2.21)	1.16 (1.06, 1.29)	<2.2e−16
Ca (mmol/L)	1.96 (1.77, 2.10)	2.20 (2.09, 2.28)	<2.2e−16
TC (mmol/L)	4.47 (3.725, 5.205)	4.99 (4.22, 5.99)	9e−12
TG (mmol/L)	1.43 (1.07, 2.00)	1.82 (1.26, 2.87)	1.2e−9
LDL (mmol/L)	2.82 (2.225, 3.59)	3.20 (2.53, 4.10)	7.5e−8
HDL (mmol/L)	0.96 (0.785, 1.19)	1.07 (0.90, 1.34)	1.2e−10
Hb (g/L)	82 (19)	131.66 (21.5)	<2.2e−16
mALB (mg/L)	1,300 (586, 2,365)	980 (309, 2,070)	0.0099

**Note:**

Normal-distribution measurement data were expressed as mean ± standard deviation, non-normal distributions are represented by median (P25%, P75%), and counting data were expressed as frequency and percentage, *p* < 0.05 was considered statistically significant. Cr, Creatinine; FIB, fibrinogen; CRP, C-reactive protein; Cys, Cystatin C; UA, serum uric acid; Urea, urea nitrogen; P, serum phosphorus; Ca, serum calcium; TC, total cholesterol; TG, triglyceride; LDL, low density lipoprotein; HDL, high-density lipoprotein; Hb, hemoglobin; mALB, microalbuminuria.

**Table 5:** Data comparison in the training and the validation group of the progress prediction model.

Indicators		Train			Validation	
		(*n* = 681)			(*n* = 291)	
	CKD-5	Early	*p* value	CKD-5	Early	*p* value
	(*n* = 364)	(*n* = 317)		(*n* = 163)	(*n* = 128)	
Gender Male, *n* (%)	210 (58%)	207 (65%)	0.05	90 (55%)	83 (65%)	0.1
Female, *n* (%)	154 (42%)	110 (35%)		73 (45%)	45 (35%)	
Age (years)	54 (42.75, 64)	50 (38, 61)	0.003	55 (42, 63)	52.5 (38, 64)	0.19
Cr (μmol/L)	598.5 (455.75, 832.75)	105 (84, 121)	<2.2e−16	584 (447.5, 796)	107.5 (89, 120)	<2.2e−16
FIB (g/L)	4.45 (3.735, 5.31)	3.69 (3.23, 4.33)	3.6e−16	4.62 (3.845, 5.45)	3.85 (3.275, 4.54)	8.9e−8
CRP (mg/L)	4.29 (2.21, 9.275)	2.9 (1.92, 5.08)	3.6e−8	3.97 (2.19, 9.015)	2.82 (1.85, 5.1075)	0.004
Cys (mg/L)	4.7 (4.0975, 5.5275)	1.42 (1.14, 1.73)	<2.2e−16	4.82 (4.115, 5.375)	1.465 (1.1875, 1.75)	<2.2e−16
UA (μmol/L)	444 (359, 557.25)	392 (325, 469)	1.4e−8	438 (350, 535)	387 (324.25, 473.75)	0.003
Urea (mmol/L)	26.315 (19.475, 33.11)	6.84 (5.54, 8.55)	<2.2e−16	26.07 (20.185, 35.065)	7.145 (5.5675, 8.7525)	<2.2e−16
P (mmol/L)	1.83 (1.5375, 2.21)	1.15 (1.05, 1.29)	<2.2e−16	1.81 (1.54, 2.195)	1.18 (1.06, 1.2925)	<2.2e−16
Ca (mmol/L)	1.95 (1.75, 2.11)	2.19 (2.09, 2.28)	<2.2e−16	1.97 (1.825, 2.09)	2.2 (2.08, 2.27)	<2.2e−16
TC (mmol/L)	4.465 (3.75, 5.17)	5 (4.14, 6.13)	2.1e−9	4.48 (3.695, 5.31)	4.93 (4.2425, 5.875)	0.001
TG (mmol/L)	1.46 (1.0775, 2.0325)	1.8 (1.26, 2.74)	4.2e−6	1.41 (1.045, 1.965)	1.685 (1.25, 2.8425)	6.5e−5
LDL (mmol/L)	2.825 (2.26, 3.5175)	3.23 (2.51, 4.13)	3.4e−6	2.8 (2.18, 3.695)	3.225 (2.5675, 4.0225)	0.007
HDL (mmol/L)	0.94 (0.77, 1.1725)	1.08 (0.89, 1.38)	8.3e−10	0.99 (0.8, 1.215)	1.03 (0.8975, 1.2825)	0.02
Hb (g/L)	82 (68, 97)	133 (119, 147)	<2.2e−16	80 (68, 93)	130 (114.75, 143.25)	<2.2e−16
mALB (mg/L)	1,305 (568, 2,360)	980 (411, 2,050)	0.02	1,260 (636, 2,385)	1,195 (335, 2,592.5)	0.341

**Note:**

Normal-distribution measurement data were expressed as mean ± standard deviation, non-normal distributions are represented by median (P25%, P75%), and counting data were expressed as frequency and percentage, *p* < 0.05 was considered statistically significant. Cr, Creatinine; FIB, fibrinogen; CRP, C-reactive protein; Cys, Cystatin C; UA, serum uric acid; Urea, urea nitrogen; P, serum phosphorus; Ca, serum calcium; TC, total cholesterol; TG, triglyceride; LDL, low density lipoprotein; HDL, high-density lipoprotein; Hb, hemoglobin; mALB, microalbuminuria.

**Table 6:** Data analysis results of CKD progress prediction model.

Variable	Scaling/Unit	OR (95% CI)	*p* value	VIF
Age	per 10-year increase	1.48 [1.12–1.95]	0.009	1.18
Uric acid (UA)	per 50 μmol/L increase	1.12 [1.04–1.21]	0.002	1.27
Phosphorus (P)	per 0.1 mmol/L increase	1.35 [1.18–1.56]	<0.001	2.04
High-density lipoprotein (HDL)	per 0.5 mmol/L increase	0.24 [0.09–0.62]	0.004	3.40
Hemoglobin (Hb)	per 10 g/L increase	0.91 [0.89–0.93]	<0.001	2.60

**Note:**

Continuous variables were rescaled using clinically meaningful units to improve interpretability of odds ratios. Variables with poor model fit (*e.g*., cystatin C, urea) or substantial multicollinearity (*e.g*., TC, LDL) were excluded during backward stepwise selection based on both statistical performance and clinical considerations. ORs represent the relative change in odds per specified unit increase. OR, Odds ratio; CI, confidence interval; Cr, Creatinine; FIB, fibrinogen; CRP, C-reactive protein; Cys, Cystatin C; UA, serum uric acid; Urea, urea nitrogen; P, serum phosphorus; Ca, serum calcium; TC, total cholesterol; TG, triglyceride; LDL, low density lipoprotein; HDL, high-density lipoprotein; Hb, hemoglobin; mALB, microalbuminuria.

### Performance and clinical utility of progression prediction model

**Figure 5:**
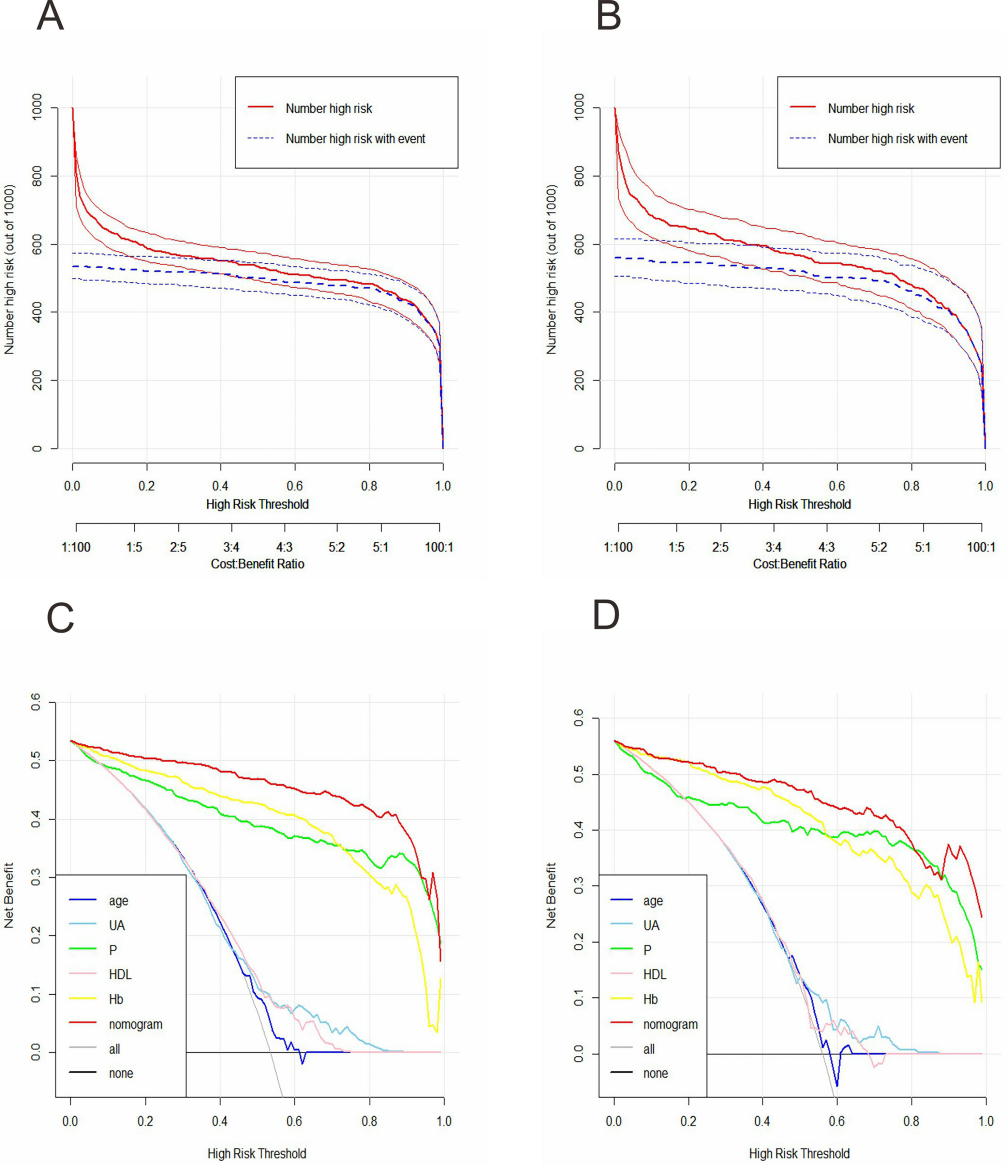
Clinical impact curves (CIC) and Decision curve analysis (DCA) for the progression prediction model of CKD in the training set and validation set. (A) CIC of the progression prediction model in the training set. X-axis: predicted risk threshold for CKD; Y-axis: number of patients identified as high-risk per 1,000; the shaded area represents the number of true positives at each threshold. (B) CIC of the progression prediction model in the validation set. (C) DCA of the progression prediction model in the training set. X-axis: threshold probability for intervention; Y-axis: net benefit per 1,000 patients; the curves compare the net benefit of using the nomogram to strategies of treating all or no patients. (D) DCA of the progression prediction model in the validation set.

We found that in the training set, the AUC was 0.984 (95% CI [0.977–0.991]), and in the validation set, it reached 0.972 (95% CI [0.957–0.987]). These values exceeded those of any single incorporated indicator (Figs. 4B and 4C). This demonstrated that the model had strong predictive capabilities. In both the training and validation sets, the progression prediction model demonstrated excellent agreement between the predicted and actual outcomes through calibration curves (Figs. 4D and 4E). In particular, for the training set, the chi-square value of the H-L test was 3.535, with a *p-*value of 0.896, while for the validation set, it was 5.566, with a *p-*value of 0.695. The DCA indicated that the net gain of the progression prediction model was more significant than that of any single factor in both the training and validation sets (Figs. 5A and 5B). The CIC revealed considerable clinical benefit (Figs. 5C and 5D).

## Discussion

As a major public health issue, CKD has caused a tremendous burden on patients due to its high incidence and low early-stage prediction rate (Goto, Iseri & Hida, 2024). A method capable of early-stage CKD prediction and assessment of progression to advanced stages may help guide clinical decision-making and reduce disease burden and complications (Chang et al., 2019). Based on routinely available clinical and laboratory indicators, this study developed an early-stage CKD prediction nomogram for individuals with preserved kidney function (G1–G2) and a progression prediction model from early-stage CKD to G5. The models demonstrated strong discriminative ability and good calibration in early-stage prediction. These models demonstrated clinical utility in identifying individuals at increased risk of early CKD, as well as in estimating the likelihood of progression to end-stage renal disease. This, in turn, created an opportunity for the early detection and intervention of such patients.

We selected 16 variables, including renal function, serum electrolyte, urinary albumin-related indices, hemoglobin, fibrinogen, metabolic indicators, and demographic characteristics indicators, which proved to be related to CKD based on clinical experience or reliable research conclusions (Sofue et al., 2020; Girndt, 2017). In our analysis, seven indicators, Cr, FIB, UA, Ca, P, TG, and Hb, were proven to be the risk factors for early CKD, and five indicators, age, UA, P, HDL, and Hb, were established to be the risk factors from early-stage CKD to G5. With the development of the disease, the abnormalities of Cr, FIB, Ca, and TG were no longer significant. Age and HDL began to appear as apparent abnormalities. Simultaneously, UA, P, and Hb exhibited marked abnormalities from the early to the late stages of the disease. This indicates a stronger correlation between these factors and the progression of the disease.

Blood creatinine mainly originates as a metabolic by-product of muscle activity and is excreted daily in urine by the kidneys. When kidney function is impaired, the creatinine produced daily cannot be fully eliminated, resulting in elevated serum creatinine concentrations (Benoit, Ciccia & Devarajan, 2020). We found that Cr was a significant risk factor for CKD (OR, 1.10, 95% CI [1.08–1.13]), similar to previous studies (Bruce & Parikh, 2023). Cr has a relatively limited ability to differentiate between the early and advanced stages (*p* = 0.995). This may be accounted for by the fact that the GFR, which is also influenced by factors such as gender and age, contributing to this observation (Wang et al., 2023).

The differential significance of predictors between the CKD risk identification model and the progression model likely reflects distinct pathophysiological processes operating at different disease stages. Predictors retained in the early-stage model tend to capture systemic inflammation, coagulation abnormalities, and early metabolic disturbances, whereas those in the progression model are more closely related to chronic metabolic burden, cardiovascular risk, and sustained renal injury. Fibrinogen (FIB), a coagulation-related indicator, was included in the early-stage prediction model. The coagulation abnormalities observed in CKD are highly complex. Numerous studies have demonstrated that patients with CKD commonly exhibit a hypercoagulable state and an increased risk of thromboembolism, which is a well-recognized complication of the disease. This phenomenon may be attributed to abnormal platelet activation, vascular endothelial dysfunction, and chronic inflammation, accompanied by increased thrombin-antithrombin complex formation (Wu et al., 2020; Kumar et al., 2019; Otto, 2018). Reduced glomerular filtration rate increases plasma fibrinogen and D-dimer levels, and serum protein loss exacerbates the risk of thrombosis. However, CKD patients are also at risk of bleeding, associated with synthesis alterations and reduced aggregation of platelets and anemia and may also explain why FIB has not emerged as a predictor of progress (Kumar et al., 2019).

Electrolyte variables are also one of the indicators reflecting glomerular filtration and tubular metabolism. Serum calcium and phosphorus have a complex regulation mechanism (Xie, Hu & Chen, 2022). The tendency of phosphorus retention has appeared as early as CKD G2 (Fan et al., 2024). In early CKD, increased FGF23 secretion promotes urinary phosphate excretion by reducing renal tubular phosphate reabsorption, thereby maintaining serum phosphorus within the normal range until advanced stages of disease. Meanwhile, FGF23-mediated inhibition of 1α-hydroxylase contributes to reduced active vitamin D synthesis and subsequent decreases in serum calcium levels (Hsu, Chen & Chen, 2020). In patients with CKD, hyperphosphatemia is associated with a higher risk of developing metabolic bone disease and experiencing cardiovascular events; it also leads to elevated mortality (Khairallah & Nickolas, 2018). Studies have also shown that elevated serum phosphorus increases the risk of death in end-stage dialysis patients. However, serum calcium analysis showed no association with a relative risk of death (Qiu et al., 2025). These observations are consistent with our findings that phosphorus was retained as a predictor in both early-stage and progression models, whereas calcium was only informative for early-stage risk identification.

Dyslipidemia is a common metabolic complication of CKD, with hypertriglyceridemia representing the most prevalent lipid abnormality. Triglyceride levels tend to increase early in CKD, largely due to impaired catabolism of very-low-density lipoproteins and chylomicrons, in secondary to reduced lipoprotein lipase activity (Kintu et al., 2023). A Mendelian randomization analysis has further suggested a causal association between elevated triglyceride levels and increased CKD risk (Zhang et al., 2020). Research indicates that HDL can alleviate oxidative stress and inflammation in chronic kidney disease. It was previously considered a protective factor against cardiovascular issues (Rysz et al., 2020). Recent evidence suggests that reduced HDL levels and dysfunctional HDL subclasses are independently associated with CKD progression, adverse outcomes, and mortality (Nam et al., 2019; Kanda et al., 2016; Navaneethan et al., 2018). In our study, HDL—but not triglycerides—was retained in the progression nomogram, suggesting that HDL may have a limited role in early disease detection but a more pronounced impact on long-term disease progression. This hypothesis warrants further investigation. These findings underscore that predictors of CKD presence and progression are not necessarily identical, but reflect different biological mechanisms at distinct disease stages.

We have also observed that older age is a risk factor for developing early stage into advanced CKD (OR = 1.48, *p* = 0.009). A study involving 430 patients with G3–G5 chronic kidney disease (CKD) found that patients aged 20–39 years and 40–64 years had a higher risk of developing end-stage renal disease (ESRD) compared to those aged 75 or older years (Chou et al., 2019), supporting the relevance of age in CKD progression risk assessment. Nevertheless, there are critiques regarding the utilization of an eGFR value below 60 L/min for diagnosing CKD in the elderly. Since eGFR typically declines with age, this early-stage prediction method has been criticized for overestimating the prevalence of CKD among the senior population (Chou & Chen, 2021). The use of traditional CKD eGFR thresholds for predicting outcomes in older adults is therefore uncertain. Our study established a multi-index combined model including age as a more sensitive formula to estimate kidney function in older adults.

In addition to the P mentioned above, UA and Hb are particularly noteworthy because they were also involved in the establishment of both the early-stage prediction model and the progress model. The pathogenesis of UA in CKD has been widely studied for a long time. UA induces endothelial dysfunction by increasing oxidative stress. In animal models, the expression of cyclooxygenase-2 (COX-2) is increased by hyperuricemia. The renin-angiotensin system activates, triggering the proliferation of vascular smooth muscle cells in the preglomerular arterioles. Consequently, glomerular hypertension develops, renal blood flow decreases, and eGFR declines (Gherghina et al., 2022; Park, Jo & Lee, 2020). Studies have shown a link between elevated serum uric acid levels and the decline in kidney function in both the general population and patients with CKD. Elevated serum uric acid is an independent risk factor for CKD (Kimura, Tsukui & Kono, 2021). Hyperuricemia raises the chance of developing and exacerbating CKD and is linked to all-cause mortality (Srivastava et al., 2018). Anemia is one of the common symptoms of CKD, and hemoglobin is an essential indicator of anemia diagnosis. The severity of anemia is closely related to CKD progression and patient survival (Pratt et al., 2022), and our models reflect this conclusion. While a decrease in hemoglobin is considered a factor in the advancement of CKD, previous studies have not identified hemoglobin as a risk indicator for the condition (Pan et al., 2022). The pathogenesis of reduced hemoglobin in CKD is still unclear, and it may be renal hypoxia caused by anemia that leads to renal injury (Pan et al., 2022).

There are some studies on CKD prediction models. For healthy population screening, Meng et al. (2022) developed a model for hidden kidney diseases, which suggested that age, gender, history of disease, proteinuria, and anemia were associated with CKD. Compared with Meng et al.’s (2022) model, we add some variables, such as coagulation and blood lipid metabolism indicators, to improve the model’s discriminative ability and calibration in early-stage prediction. The population-specific predictive models are also developed for the risk of developing ESRD. A Singapore study of low-risk people from different ethnic groups in the Asian population shows that the best end-stage renal failure prediction models include age, gender, eGFR, and proteinuria, emphasizing that increasing race variables can improve the model (Lim et al., 2019). Importantly, the proposed models are not intended to replace KDIGO-based risk stratification criteria, but rather to complement standard evaluation by providing a multivariable framework for CKD-related risk stratification using routinely available laboratory data, rather than to establish or replace a formal diagnosis of CKD. The differential significance of predictors between the diagnostic and progression models likely reflects distinct pathophysiological stages of CKD. Biomarkers such as fibrinogen and calcium may be more sensitive to early systemic inflammation and mineral metabolism disturbances that facilitate CKD detection, whereas factors such as HDL cholesterol appear to be more closely related to long-term metabolic dysregulation and cardiovascular burden, which are known drivers of CKD progression rather than initial diagnosis. Extreme ORs were driven by unit scaling rather than by biological implausibility.

This study has several limitations. First, as a retrospective cohort study, causal relationships between predictors and CKD outcomes cannot be definitively established. Second, external validation was not performed due to data limitations. Although bootstrap internal validation demonstrated good performance, the absence of external validation may increase overfitting risk and limit generalizability. Third, while CKD classification followed KDIGO 2023/2024 guidelines, urinary albumin was measured as concentration rather than albumin-to-creatinine ratio or 24-h excretion, potentially leading to misclassification in early-stage CKD (G1–G2). Fourth, comorbidities such as diabetes and hypertension were not fully accounted for, which may introduce bias. Finally, the study was conducted at a single center with a relatively homogeneous population, limiting broader applicability.

Future work will focus on multicenter validation, inclusion of more heterogeneous populations, integration of additional covariates (medications, lifestyle, comorbidities), and prospective studies to clarify temporal and causal relationships, thereby enhancing model robustness and clinical utility.

## Conclusion

In summary, this study proposes laboratory-based nomogram models for CKD-related risk assessment and progression evaluation. While the models exhibited favorable performance within the study cohort, their application should be interpreted within the context of the study’s limitations. Prospective, multicenter studies incorporating standardized kidney damage markers are warranted to further validate and refine these models before broader clinical implementation.

## Supplemental Information

10.7717/peerj.20931/supp-1 Supplemental Information 1 Anonymized raw data.

10.7717/peerj.20931/supp-2 Supplemental Information 2 Original data of the progression.

10.7717/peerj.20931/supp-3 Supplemental Information 3 STROBE checklist.

10.7717/peerj.20931/supp-4 Supplemental Information 4 All original data.

## Additional Information and Declarations

### Data Availability

The following information was supplied regarding data availability:

The raw measurements are available in the Supplemental Files.
